# Application of Hepatic Venous Pressure Gradient to Predict Prognosis in Cirrhotic Patients with a Low Model for End-stage Liver Disease Score

**DOI:** 10.3390/diagnostics10100805

**Published:** 2020-10-10

**Authors:** Young Chang, Ki Tae Suk, Soung Won Jeong, Jeong-Ju Yoo, Sang Gyune Kim, Young Seok Kim, Sae Hwan Lee, Hong Soo Kim, Seong Hee Kang, Soon Koo Baik, Dong Joon Kim, Moon Young Kim, Jae Young Jang

**Affiliations:** 1 Department of Internal Medicine, Institute for Digestive Research, Digestive Disease Center, Soonchunhyang University College of Medicine, Seoul 04401, Korea; chyoung86@gmail.com (Y.C.); jeongsw@schmc.ac.kr (S.W.J.); 2 Department of Internal Medicine, Institute for Liver and Digestive Diseases, Hallym University College of Medicine, Chuncheon 24252, Korea; ktsuk@hallym.ac.kr (K.T.S); djkim@hallym.ac.kr (D.J.K.); 3 Department of Internal Medicine, Soonchunhyang University College of Medicine, Bucheon 14584, Korea; puby17@naver.com (J.-J.Y.); mcnulty@schmc.ac.kr (S.G.K.); dr.yskim@gmail.com (Y.S.K.); 4 Department of Internal Medicine, Soonchunhyang University College of Medicine, Cheonan 31151, Korea; naozman@gmail.com (S.H.L.); khskhs@schmc.ac.kr (H.S.K.); 5 Department of Internal Medicine, Yonsei University, Wonju College of Medicine, Wonju 26426, Korea; shkang0114@gmail.com (S.H.K.); baiksk@yonsei.ac.kr (S.K.B.)

**Keywords:** HVPG, low-MELD, liver transplantation

## Abstract

Background/aim: We aimed to derive a model representing the dynamic status of cirrhosis and to discriminate patients with poor prognosis even if the Model for End-Stage Liver Disease (MELD) score is low. Methods: This study retrospectively enrolled 700 cirrhotic patients with a MELD score of less than 20 who underwent hepatic venous pressure gradient (HVPG) measurement. A model named H6C score (= HVPG + 6 × CTP score) to predict overall survival was derived and internal and external validations were conducted with the derivation and validation cohorts. Results: The H6C score using the HVPG was developed based on a multivariate Cox regression analysis. The H6C score showed a great predictive power for overall survival with a time-dependent AUC of 0.733, which was superior to that of a MELD of 0.602. In patients with viral etiology, the performance of the H6C score was much improved with a time-dependent AUC of 0.850 and was consistently superior to that of the MELD (0.748). Patients with an H6C score below 45 demonstrated an excellent overall survival with a 5-year survival rate of 91.5%. Whereas, patients with an H6C score above 64 showed a dismal prognosis with a 5-year survival rate of 51.1%. The performance of the H6C score was further verified to be excellent in the validation cohort. Conclusion: This new model using the HVPG provides an excellent predictive power in cirrhotic patients, especially with viral etiology. In patients with H6C above 64, it would be wise to consider early liver transplantation to positively impact long-term survival, even when the MELD score is low.

## 1. Introduction

Various models have been suggested to predict the prognosis of patients with cirrhosis. The Model for End-Stage Liver Disease (MELD), initially developed to predict the survival of patients undergoing the transjugular intrahepatic portosystemic shunt procedure [1] has been broadly validated to predict both the short-term and long-term mortalities in a heterogeneous population of patients with cirrhosis [2,3,4,5]. Accordingly, liver allocation for liver transplantation has been based on the MELD or MELD-sodium score, and MELD is considered highly accurate in predicting liver-related mortality before liver transplantation [3,6]. The MELD along with the Child-Turcotte-Pugh (CTP) scoring system is most commonly used to determine the extent of hepatic dysfunction and prognosis; however, direct evidence regarding the dynamic state of cirrhosis is not indicated in both models.

Portal hypertension (PHT) is a common clinical syndrome induced by cirrhosis and is responsible for the major complications of cirrhosis such as ascites, hepatic encephalopathy, and variceal bleeding [7]. PHT is defined as a pathological increase of the portal venous pressure gradient between the portal vein and inferior vena cava (IVC) [7,8]. Since Myers and Taylor initially used the wedged hepatic venous pressure by occlusive hepatic vein catheterization to estimate portal venous pressure [9], safe, reproducible, and less invasive methods to estimate the portal venous pressure gradient have been developed [10]. Several indicators of portal hypertensive syndrome have been developed; of these, the measurement of hepatic venous pressure gradient (HVPG) is considered the most direct and accessible approach [11]. Physicians can perform direct measurement of the portal and hepatic venous pressures under endoscopic ultrasound (EUS) guidance using a new device recently approved by the US FDA. However, EUS-guided measuring is a newly developed method that needs to be validated, and further researches on the clinical significance of the EUS-guided measured portal pressure are necessary. Still, HVPG has been regarded as a surrogate indicator of PHT and widely used for risk stratification, prognostic assessment, and monitoring of treatment responses in patients with chronic liver disease [10,11]. A recent study has also suggested that HVPG is beneficial in assessing the severity of PHT and treatment responses in real clinical practice [12].

Currently, re-evaluation of the role of PHT indexes combined with conventional scoring systems has been suggested considering that PHT had been regarded as the third parameter most frequently found to be a significant predictor of survival in cirrhosis [11,13]. Since mounting evidence has demonstrated that cirrhosis is neither static nor relentlessly progressive, but rather dynamic and sometimes bidirectional, the stratification of cirrhotic patients based on their hemodynamic status in relation with PHT is required [14]. When patients were divided into several categories according to the MELD score, a 1-year mortality within each category was demonstrated to be higher in patients with PHT-related complications than in those without PHT-related complications [15]. Therefore, we aimed to derive a model representing the dynamic status of cirrhosis to discriminate patients with poor prognosis even with a low MELD score.

## 2. Method

### 2.1. Study Population

This multicenter study recruited consecutive patients with cirrhosis who underwent an HVPG measurement in four academic hospitals in Korea. A derivative cohort (from four hospitals from January 2008 to June 2013) and validation cohort (from two hospitals from July 2013 to June 2016) were established to derive and validate predictive models.

Baseline cirrhosis was diagnosed based on imaging findings indicative of cirrhosis such as surface nodularity, blunted edge, hypertrophied left lobe and atrophied right lobe, and splenomegaly in combination with clinical signs of PHT including ascites, esophageal or gastric varices, and collateral vessel formation. HVPG was measured on clinical demands to determine the basal portal pressure prior to variceal bleeding prophylaxis or to consider the presence of PHT as an adjunct to the diagnosis of cirrhosis. Patients were excluded if they met any of the following criteria: Recent variceal bleeding within 2 weeks, jaundice with a total bilirubin level above 5 mg/dL, acute or chronic kidney injury with a serum creatinine level above 1.5 times the upper normal limit, high MELD score greater than 20, portal vein thrombosis, prior diagnosis of any malignancies, and loss of follow-up within 28 days. All of the patients were not active drinkers, defined as consuming alcohol more than 140 g/week for women and 210 g/week for men. In particular, for patients with alcoholic cirrhosis, alcohol abstinence for at least 6 months prior to HVPG measurement was considered eligible for this study. Patients with hepatitis B were in an inactive state, whereas, not all the patients with hepatitis C had been treated to cure because direct-acting antivirals were not commonly used in Korea during the study period from 2008 to 2013. However, patients who had active hepatitis or acute decompensation caused by hepatitis C virus were excluded by the general principle of enrollment to this study.

A total of 700 compensated cirrhotic patients with PHT were finally included: 566 for the derivative cohort and 134 for the validation cohort. Medical records of the study population were retrospectively reviewed, and data were collected including various laboratory findings, individual medical status, HVPG, and overall survival (OS).

### 2.2. Hepatic Venous Pressure Gradient Measurement 

A six-French balloon-tip catheter (Arrow International, Cleveland, OH, USA) was inserted into the right hepatic vein via the right jugular vein. Free hepatic venous pressure (FHVP) was measured within 5 cm from the junction of the right hepatic vein and IVC, and at the same location, the wedged hepatic venous pressure was measured after balloon inflation. Each measurement was repeated three times, and the mean values were reported. HVPG was calculated by obtaining the difference between the free and wedged (ballooned) hepatic venous pressure. The procedures were performed based on the principle that the difference between FHVP and IVC pressure should not be significant and measured values were reproducible [10,16]. If the above conditions were not satisfied, the wedged position of the catheter was reconfirmed.

Any vasoactive drugs that can affect the patient’s hemodynamics were discontinued at least 72 h before the procedure. All procedures were performed by well-experienced interventional radiologists or hepatologists.

### 2.3. Statistical Analysis

The baseline characteristics were compared between groups using the independent-samples t-test for continuous variables and chi-squared test for categorical variables. OS was estimated using the Kaplan-Meier method and compared using the log-rank test. The independent risk factors for death were identified using the univariate and multivariate Cox proportional hazards model. The performance of the models to predict OS was analyzed graphically using the time-dependent receiver operating characteristics (ROC) curve and numerically using the time-dependent area under the curve (AUC).

Differences with a *p*-value less than 0.05 were considered statistically significant. All statistical analyses were performed using R version 3.5.3 (R Foundation for Statistical Computing, Vienna, Austria). The survival ROC package and time ROC package were used in R to perform time-dependent ROC and AUC analysis.

### 2.4. Ethical Consideration

This study followed the recent ethical guidelines of the World Medical Association Declaration of Helsinki and was approved by the Institutional Review Board (IRB) of Soonchunhyang University Seoul Hospital (IRB no. 2013-12-005; 24/12/2013). All the included patients provided written informed consent for inclusion in the study. Medical records of the study population were fully anonymized and de-identified before analysis.

## 3. Results

### 3.1. Baseline Characteristics

Table 1 and Supplementary Table S1 show the baseline characteristics of the overall study population. The mean age was 52.55 years, and the majority of the participants were men. Among the study population, 89.6% had Child-Pugh class A or B, and the mean value of MELD was 10.12. The mean age and baseline liver function represented by Child-Pugh score and MELD did not differ significantly between the derivation and validation cohorts. However, the validation cohort showed a lower level of HVPG and higher proportion of Child-Pugh class of patients with a favorable platelet count and MELD score than the derivation cohort. 

Of the 566 patients in the derived cohort, 84 patients died during the study period. In addition, two patients underwent liver transplantation due to hepatic encephalopathy (10 months after enrollment) and hepatic failure (20 months after enrollment), and they were counted as death events. The majority of deaths (68 out of 86, 79.1%) were liver related mortalities, mainly due to hepatic failure or variceal bleeding. Of the rest, 10 died from infection and five died from cerebral hemorrhage, which could potentially be promoted by decreased liver function. In the validation cohort, five out of 134 patients died during the study period. The mean follow-up time was 25.05 ± 18.01 months in the derived cohort and 26.40 ± 17.02 months in the validation cohort.

### 3.2. Development of a Novel Risk Scoring Model from The Derivation Cohort

According to the univariate analysis, the serum level of albumin, total bilirubin, alanine aminotransferase, prothrombin time, CTP score, MELD, and HVPG were significantly associated with the risk of death of the study population (Table 2). On the contrary, age, platelet count, and serum creatinine level were not revealed as risk factors. As a result, CTP score and HVPG, which represented those identified prognostic factors comprehensively, were included in multivariate analysis. Multivariate analysis showed that the CTP score (hazard ratio (HR) = 1.32, 95% confidence interval (CI) = 1.18–1.48, *p* < 0.001) and HVPG (HR= 1.07, 95% CI = 1.02–1.12, *p* = 0.003) were independently associated with the risk of death. Several adjustments were conducted considering the model’s performance and user convenience, and finally, a novel risk scoring model was derived as follows: H6C score = HVPG + 6 × CTP score.

### 3.3. Impact of The H6C Score In Predicting The Overall Survival (OS) Of Cirrhotic Patients

**Figure 1:**
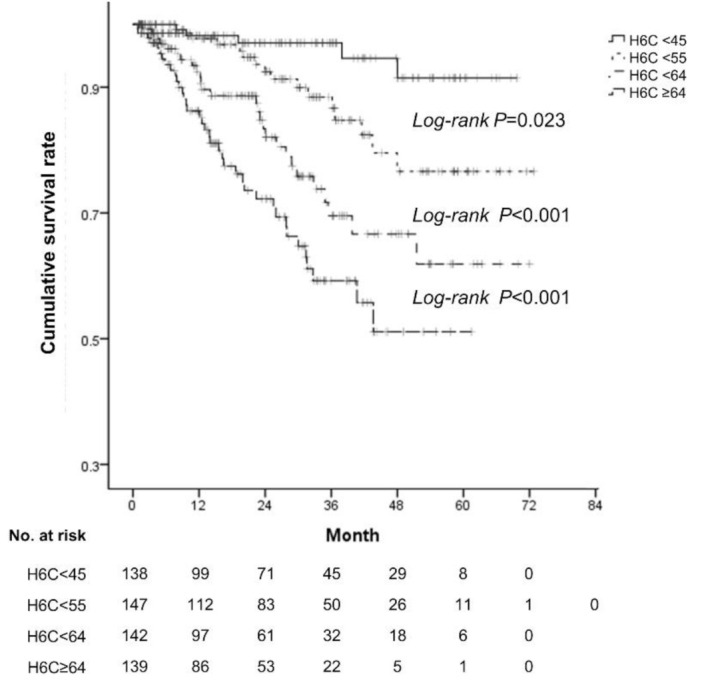
The cumulative survival rates according to the H6C score in the derivation cohort. The survival rates significantly differed according to quartiles of the H6C score (shown as a continuous line and various dashed line).

The median H6C score in the derivation cohort was 54 (range, 36–102). When patients were stratified into four groups by quartiles of the H6C score (H6C < 45, 45 ≤ H6C < 55, 55 ≤ H6C < 64, and 64 ≤ H6C), each group of patients showed significantly different baseline liver function and HVPG with statistically similar demographic characteristics (Supplementary Table S2). Moreover, the differences of OS were statistically significant between each of the four groups (all log-rank *p* < 0.05, Figure 1). If the H6C score was less than 45, the lowest quartile, the 5-year survival rate was 91.5%. On the contrary, if the H6C score was greater than 64, the highest quartile, the 5-year survival rate was only 51.1%.

### 3.4. Prognostic Power Of The Models For Predicting OS

**Figure 2:**
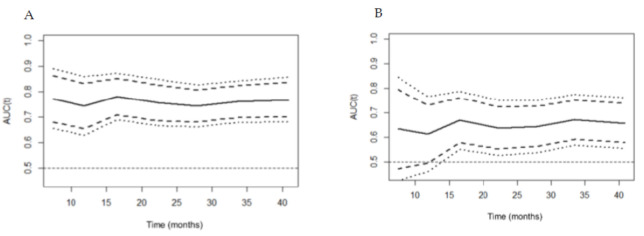
The curve of time-dependent area under the curve (AUC) for predicting the overall survival of the H6C score (**A**) and the model for end-stage liver disease (MELD) (**B**). The continuous line represents time-dependent AUC, and the dashed line and dotted line show 95% and 99% confidence internal, respectively. The AUCs of the H6C score were consistently higher than those of the MELD over time.

Based on the time-dependent ROC curves, the H6C score represented a great discrimination function in predicting OS with an area under the time-dependent ROC of 0.733, which was higher than that of MELD (0.602; Supplementary Figure S1). When analyzed over time, the time-dependent AUC of the H6C score was consistently higher than that of MELD, ranging from 0.744 to 0.773 over time (Figure 2 and Table 3). In predicting the 1-, 3-, and 5-year survival rates, the H6C score maintained an excellent performance with AUCs of 0.754 (95% CI= 0.667–0.841), 0.735 (95% CI= 0.679–0.790), and 0.712 (95% CI= 0.658–0.767), respectively (Supplementary Figure S2).

### 3.5. Prognostic Power of The H6C Score For Predicting OS In Patients With Viral Etiology

**Figure 3:**
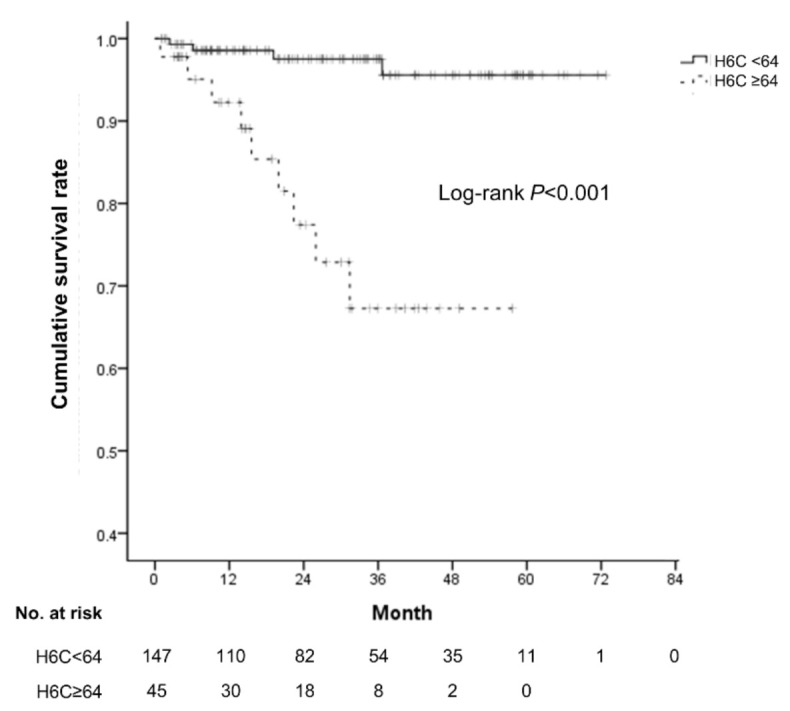
The cumulative survival rates according to the H6C score in patients with viral etiology. Patients with a low H6C score (shown as a continuous line) showed significantly higher survival rate than those with a high H6C score (shown as a dashed line).

Among the 566 patients in the derivative cohort, 192 had viral etiology of cirrhosis. The viral etiology group consisted mainly of those with chronic hepatitis B (*n* =166, 86.5%), and most of the nonviral etiology group consisted of those with alcoholic liver disease (*n* =341, 91.2%). The viral etiology group showed a lower CTP score and HVPG level, which means that they had a relatively preserved liver function overall (Supplementary Table S3). In the viral etiology subgroup, the OS differed significantly according to the H6C score with a cutoff value of 64 (log-rank *p* < 0.001, Figure 3). If the H6C score was less than 64, the 5-year survival rate was 95.6%, whereas, for patients with an H6C score greater than 64, the 5-year survival rate was reduced to 67.6%.

**Figure 4:**
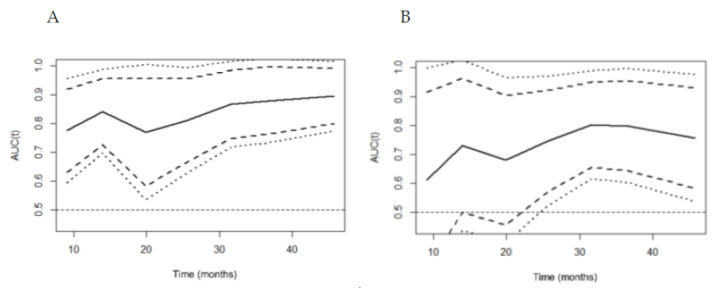
The curve of time-pendent area under the curve (AUC) for predicting the overall survival of the H6C score (**A**) and the model for end-stage liver disease (MELD) (**B**) in patients with viral etiology. The continuous line represents time-dependent AUC, and the dashed line and dotted line show 95% and 99% confidence internal, respectively. The AUCs of the H6C score were consistently higher than those of the MELD over time.

The performance of the H6C score was improved to the area under the time-dependent ROC of 0.850 over that in the entire derivation cohort. The area under the time-dependent ROC of MELD was 0.748, which was better than that in the entire derivation cohort but still lower compared to that of the H6C score in the viral etiology subgroup. When analyzed according to time, the time-dependent AUC of the H6C score increased gradually from 0.776 to 0.896 over time and was consistently higher than that of MELD (Figure 4 and Table 3). In predicting the 1-, 3-, and 5-year survival rates, the H6C score maintained an excellent performance with AUCs of 0.809 (95% CI=0.649–0.969), 0.823 (95% CI=0.702–0.945), and 0.805 (95% CI=0.686–0.923), respectively.

### 3.6. External Validation of The H6C Score in Predicting OS Of Cirrhotic Patients

The performance of H6C score was further evaluated in the validation cohort. There was a significant difference in OS according to the H6C score (log-rank *p* = 0.02, Supplementary Figure S3). When the H6C score was less than 64, the 5-year survival rate was 99%, while in patients with an H6C score greater than 64, the 5-year survival rate was reduced to 68.6%. The time-dependent AUC of the H6C score ranged from 0.814 to 0.890 over time, reflecting an excellent prognostic power, and was consistently higher than that of MELD (Supplementary Figure S4 and Table S4). In predicting the 1-, 3-, and 5-year survival rates, the H6C score maintained an excellent performance with AUCs of 0.788 (95% CI=0.399–1), 0.861 (95% CI=0.681–1), and 0.861 (95% CI=0.681–1), respectively.

## 4. Discussion

This large-scale multicenter cohort study was designed to demonstrate the impact of PHT on OS in cirrhotic patients with a low MELD score. This study primarily aimed to develop a model representing the dynamic status of cirrhosis and to discriminate patients with dismal prognosis even with a low MELD score. The H6C score was derived using the HVPG and CTP score, and it showed an excellent performance to predict OS, which was superior to that of MELD. In patients with viral etiology, the predictive power of the H6C score was further enhanced and consistently superior to that of MELD. Additionally, the excellent performance of the H6C score was further validated in the validation cohort.

The impact of PHT on the prognosis of cirrhotic patients is well established [11,13]. Several efforts combining PHT to the MELD has been made, considering that the MELD does not directly reflect PHT [2,11,15,17]. Although adding to the MELD common complications of PHT such as ascites, variceal bleeding, spontaneous bacterial peritonitis, and hepatic encephalopathy had improved the predictive power of the model to a minimum in the initial validation [2,3], other studies have shown that the inclusion of hepatic encephalopathy or moderate ascites improves the prognostic value of the MELD [15,17]. However, each individual PHT-related complication reflects only a single aspect but not the unique hemodynamics of the portal hypertensive syndrome as a whole [11]. In this context, HVPG can be potentially used as a variable that reflects PHT properly because it is a directly measured portal pressure expressed by numeric scale. Several studies have confirmed HVPG as a prognostic indicator of death in cirrhosis [11,18]. 

The prognostic value of HVPG in patients with cirrhosis is highly dependent on the accuracy of the technique [16]. It has been empirically suggested that HVPG can be measured using the IVC pressure instead of FHVP as a reference value when the difference between the FHVP and IVC pressure exceeds 2 mmHg [19]. However, there was no data supporting this recommendation, and rather, a recent study has shown that regardless of the degree of difference between FHVP and IVC pressure, HVPG using FHVP is accurate in assessing the prognosis of cirrhotic patients [20].

The MELD has been widely used to predict the prognosis in cirrhotic patients, but information about the role of the MELD in patients with early-stage liver disease is insufficient [15]. Theoretically, patients with a low MELD score have relatively low predicted risks of 90-day mortality. According to a study of liver transplant candidates, the observed 3-month mortality rates were 1.9% and 6% in patients with MELD scores less than 9 and 20, respectively [6]. However, it only reports the short-term mortality rates, and long-term outcomes for patients with a low MELD score are not well-delineated [21]. Moreover, the MELD may not accurately predict the mortality rate of certain individuals, specifically those with clinically significant complications mainly resulting from PHT. Atiemo et al. reported that the 1-year predicted mortality rate was greater than 14% in selected patients who had several complications including hepatic hydrothorax regardless if their initial MELD score was less than 15 [22]. Patients with PHT-related complications do not necessarily have a high MELD score; therefore, they may be downstaged and will not be prioritized for liver transplantation despite a substantial risk of mortality [21,23]. It is suggested that expanded models using certain non-MELD variables can more accurately predict and risk stratifying patients with a low MELD score [21].

We developed a new risk predicting model named the H6C score based on the clinical significance of PHT, the limitation of the MELD, and the unmet need for predicting and stratifying long-term prognosis in patients with a low MELD score. The H6C score consists of CTP score and HVPG, which represent the stage of cirrhosis in both clinical and hemodynamic contexts, resulting in an elaborate prediction of OS [14]. This score is optimized in prognosticating patients with a low MELD score since PHT-related clinical decompensation has a substantial influence on the prognosis, specifically in patients with a low MELD score. If the H6C score was less than 45, the 5-year survival rate was 91.5%; however, if the H6C score was greater than 64, the 5-year survival rate was merely 51.1%, although the initial MELD score was less than 20. In addition, this model would be beneficial for risk stratification for rebleeding prophylaxis, because this model consists of both clinical and hemodynamic information of cirrhotic patients [24]. A future study using this model in predicting variceal rebleeding would be valuable.

In this study, the performance to predict OS of the H6C score was superior to the MELD, and also better than MELD-sodium and the Child-Pugh score (data were not shown). The predicting power was further enhanced in patients with viral etiology (i.e., hepatitis B virus and hepatitis C virus infections). Among the study population, most of the patients with nonviral etiology have alcoholic cirrhosis. In these alcoholic cirrhosis patients, the level of HVPG varies significantly depending not only on the severity of cirrhosis but also on alcohol consumption and superimposed alcoholic hepatitis. Basically, a dense pericellular and perivenular collagen deposition, a typical pattern of hepatic fibrosis in alcoholic cirrhosis, leads to marked distortion of hepatic architecture, resulting in impaired blood flow in the sinusoid [25,26]. Additional factors such as acute exposure to alcohol and systemic inflammation further attenuate the intrahepatic blood flow [27,28]. On the contrary, if the patients abstain completely from alcohol for several weeks, a significant reduction in HVPG can be achieved [29]. In such alcoholic cirrhotic patients, it may be relatively inaccurate to predict the long-term outcomes with a single initial HVPG value because the HVPG level is highly variable and sensitive to various factors such as alcohol consumption and systemic inflammation.

This study has several limitations. First, because of the retrospective nature of the study, the information regarding various baseline variables such as retroperitoneal portal systemic shunts and other outcomes including the development of acute decompensation and other liver related events than OS was not available. Liver related events including acute decompensation are significant because it may ultimately determine the patient’s mortality. Accordingly, OS is among the most valuable and difficult outcome to be assessed. In addition, the data regarding repeated HVPG measurements in patients with variceal bleeding were not available. It has been reported that the hemodynamic response (HVPG reduction to <12 mmHg or by >20% from baseline) to pharmacological therapy may achieve an improvement on survival in patients with PHT [30,31,32,33]. Reducing portal pressure in patients with variceal bleeding may contribute to prevent rebleeding and further decompensation of cirrhosis, thus improving overall survival. However, since only 6% of patients in this study experienced variceal bleeding prior to HVPG measurement and the measurement was not performed at the time of bleeding, HVPG monitoring was not generally necessary. Second, the study population could appear to be heterogeneous because we included selected patients with ascites and/or hepatic encephalopathy based on medical records that the physician determined that those complications were controllable and that the patient was in a chronic stable state rather than an acute decompensated state. However, on the contrary, even if the patient’s MELD score is low, the degree of PHT varies considerably, indicating that the patient’s spectrum is quite broad. Further well-designed prospective studies are needed to make the study population more homogeneous and to evaluate outcomes in a variety of ways other than overall survival. Third, this study was conducted on patients clinically diagnosed with cirrhosis according to the typical imaging findings along with signs of PHT, rather than histologically confirmed. Although some non-cirrhotic patients might have been enrolled in the study population, it would not have had a significant impact on predicting patients with poor prognosis. Fourth, since this cohort study included data from three large-volume hospitals in Korea, HVPG was measured by different operators at each medical center, and it was impossible to assess the interoperator variability. However, all the operators were highly experienced and qualified experts. Finally, the H6C score is a newly developed model based on the regional experiences in specific medical centers in South Korea. Moreover, HVPG measurement is an invasive procedure and requires skilled techniques and equipment, so it is not always possible in all medical centers; hence, it may lack generalizability. Further validation is required to evaluate whether the H6C score can predict OS in other cohorts or other various etiologies of cirrhotic patients.

In conclusion, the H6C score showed an excellent performance for predicting OS in cirrhotic patients with a low MELD score, and its predictive power was superior to that of the MELD, a current standard prognostic model. In patients with viral etiology, the performance of the H6C score was further enhanced. When the H6C score is higher than 64, it would be better to consider early liver transplantation for long-term survival even if the MELD score is low.

## Figures and Tables

**Table 1:** Baseline characteristics of the overall study population.

	Total (*n* =700)	Derivation Cohort (*n* =566)	Validation Cohort (*n* =134)	*p*-Value
Age (year)	52.55 ± 10.43	52.54 ± 9.71	52.59 ± 13.1	0.968
Male, no. (%)	521 (74.4)	435 (76.9)	86 (64.2)	<0.001 *
Platelet count	120.07 ± 74.37	117.27 ± 72.65	131.89 ± 80.47	0.056
Albumin (g/dL)	3.3 (2.9, 3.8)	3.3 (2.9, 3.7)	3.4 (2.92, 4)	0.059
Total bilirubin (mg/dL)	1.57 ± 1.25	1.61 ± 1.13	1.41 ± 1.66	0.194
AST (U/L)	52 (36, 76)	51 (36, 75)	55.5 (35, 84.75)	0.539
ALT (U/L)	27 (17, 49.25)	26 (17, 43.75)	35.5 (20.25, 82.25)	<0.001 *
Prothrombin time (INR)	1.21 ± 0.23	1.21 ± 0.24	1.21 ± 0.22	0.891
Creatinine (mg/dL)	0.78 ± 0.21	0.76 ± 0.21	0.83 ± 0.24	0.002 *
Ascites, no. (%)				0.004 *
None	376 (53.71%)	300 (53%)	76 (56.72%)	
Small	231 (33%)	200 (35.34%)	31 (23.13%)	
Moderate	93 (13.29%)	66 (11.66%)	27 (20.15%)	
Hepatic encephalopathy, no. (%)				<0.001 *
None	648 (92.57%)	528 (93.29%)	120 (89.55%)	
Grade 1–2	32 (4.57%)	29 (5.12%)	3 (2.24%)	
Grade 3–4	20 (2.86%)	9 (1.59%)	11 (8.21%)	
Child-Pugh score	7 (5, 8)	7 (6, 8)	6 (5, 8)	0.213
Child-Pugh class				0.163
A	336 (48%)	263 (46.47%)	73 (54.48%)	
B	291 (41.57%)	245 (43.29%)	46 (34.33%)	
C	73 (10.43%)	58 (10.25%)	15 (11.19%)	
MELD	10.12±3.24	10.2±3.25	9.82±3.17	0.217
MELD-sodium	10.56±4.65	10.71±4.67	9.91±4.55	0.069
HVPG (mmHg)	14.48±5.20	13.77±5.03	14.75±5.08	0.030 *

Data were presented as mean ± SD or median with IQR. AST: Aspartate aminotransferase; ALT: Alanine aminotransferase; MELD: Model for end-stage liver disease; HVPG: Hepatic venous pressure gradient; SD: Standard deviation; IQR: Interquartile range. * *p* < 0.05

**Table 2:** Univariate and multivariate regression analysis using the Cox proportional hazards model to predict the risk of death.

	Univariate Analysis		Multivariate Analysis	
	Hazard Ratio (95% CI)	*p*-Value	Hazard Ratio (95% CI)	*p*-Value
Sex	0.75 (0.44–1.27)	0.287		
Age	1.01 (0.99–1.03)	0.475		
Platelet count	1.00 (0.996–1.003)	0.790		
Albumin	0.32 (0.22–0.47)	<0.001 *		
Total bilirubin	1.41 (1.20–1.67)	<0.001 *		
AST	0.998 (0.993–1.002)	0.292		
ALT	0.99 (0.985–1.000)	0.042 *		
Prothrombin time	3.86 (1.83–8.12)	<0.001 *		
Creatinine	0.95 (0.34–2.67)	0.916		
Child-Pugh score	1.42 (1.28–1.57)	<0.001 *	1.32 (1.18–1.48)	<0.001 *
MELD	1.14 (1.08–1.21)	<0.001 *		
HVPG	1.12 (1.07–1.16)	<0.001 *	1.07 (1.02–1.12)	0.003 *

CI: Confidence interval; AST: Aspartate aminotransferase; ALT: Alanine aminotransferase; MELD: Model for end-stage liver disease; HVPG: Hepatic venous pressure gradient. * *p* < 0.05

**Table 3:** Estimated time-dependent AUC of the H6C score and the MELD.

Subject	Time (months)	H6C		MELD	
		AUC	95% CI	AUC	95% CI
Derivation cohort	7.43	0.773	0.682–0.865	0.636	0.473–0.798
	11.865	0.744	0.655–0.833	0.614	0.497–0.731
	22.385	0.757	0.686–0.828	0.639	0.553–0.725
	33.465	0.761	0.698–0.824	0.672	0.592–0.752
	40.87	0.769	0.701–0.837	0.658	0.579–0.738
Viral etiology of derivation cohort	9.09	0.776	0.632–0.920	0.612	0.308–0.916
	13.939	0.842	0.727–0.958	0.732	0.500–0.964
	25.585	0.812	0.666–0.957	0.747	0.571–0.923
	36.658	0.880	0.764–0.996	0.799	0.644–0.954
	45.778	0.896	0.800–0.993	0.758	0.585–0.930

AUC: Area under the receiver operating characteristic curve; MELD: Model for end-stage liver disease; CI: Confidence interval.

## Supplementary Materials

The following are available online at https://www.mdpi.com/2075-4418/10/10/805/s1.
