# Supplementary material for: Application of Hepatic Venous Pressure Gradient to Predict Prognosis in Cirrhotic Patients with a Low Model for End-stage Liver Disease Score
Source: Diagnostics (Basel). 2020 Oct 10;10(10):805. doi: 10.3390/diagnostics10100805 (PMC7599657; doi:10.3390/diagnostics10100805)
Supplement: Supplementary file 1 [file diagnostics-10-00805-s001.pdf]

diagnostics

MDPI

Supplement

A B

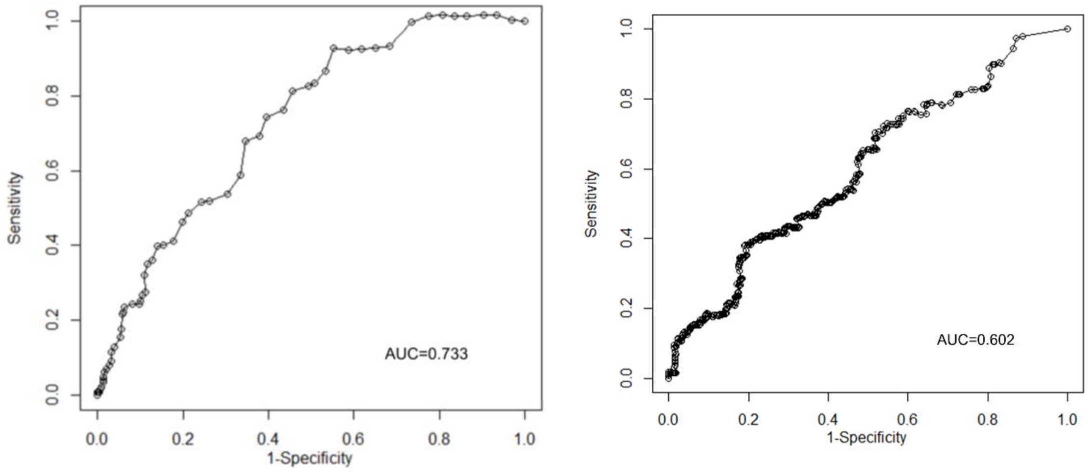

Supplementary Figure 1. The time-dependent receiver operating characteristic (ROC) curve for predicting overall survival of the H6C score (A) and the Model for End-Stage Liver Disease (MELD) (B). The area under the time-dependent ROC of the H6C score was higher than that of the MELD.

A **B**

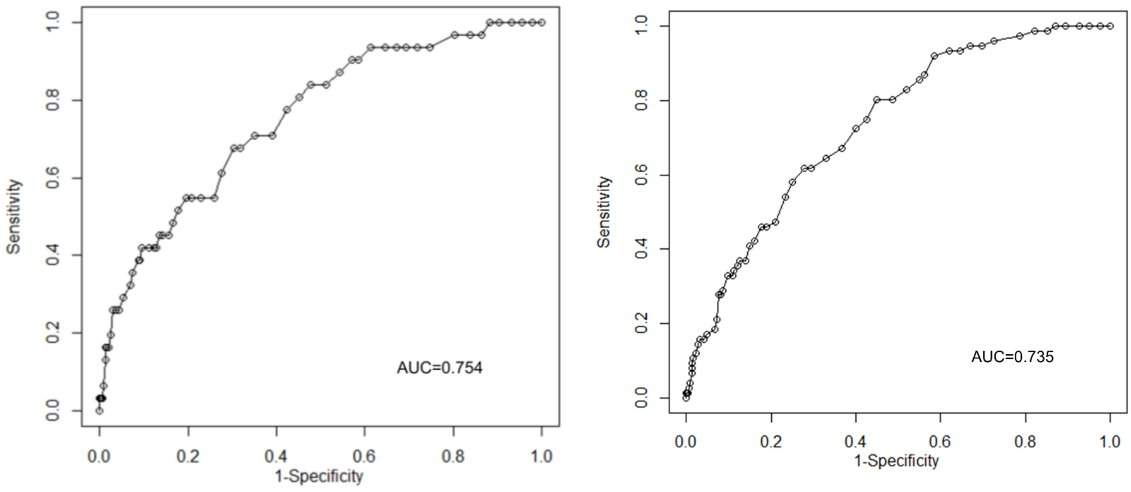

C

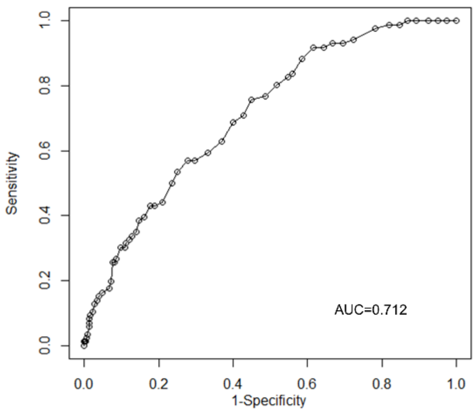

Supplementary Figure 2. The receiver operating characteristic curves for predicting the 1-year (A), 3-year (B) and 5-year (C) survival rate of the H6C score.

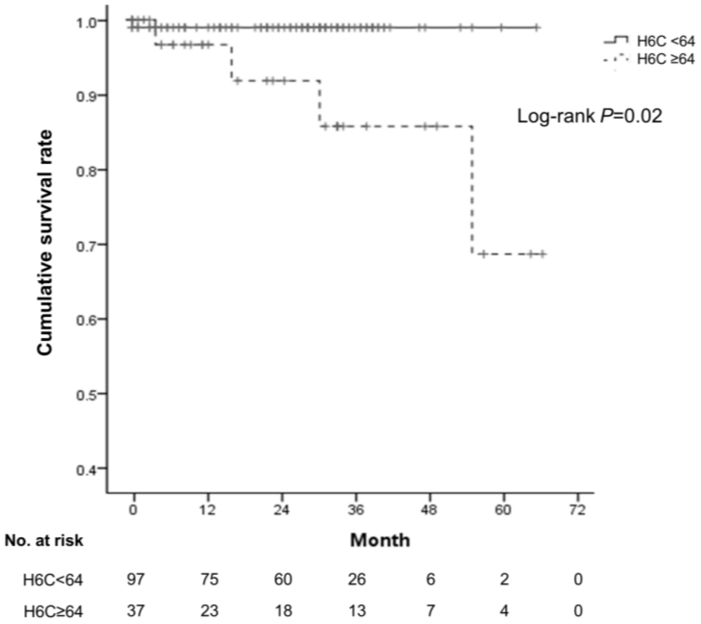

Supplementary Figure 3. The cumulative survival rates according to the H6C score in the validation cohort. Patients with a low H6C score (shown as a continuous line) showed significantly higher survival rate than those with a high H6C score (shown as a dashed line).

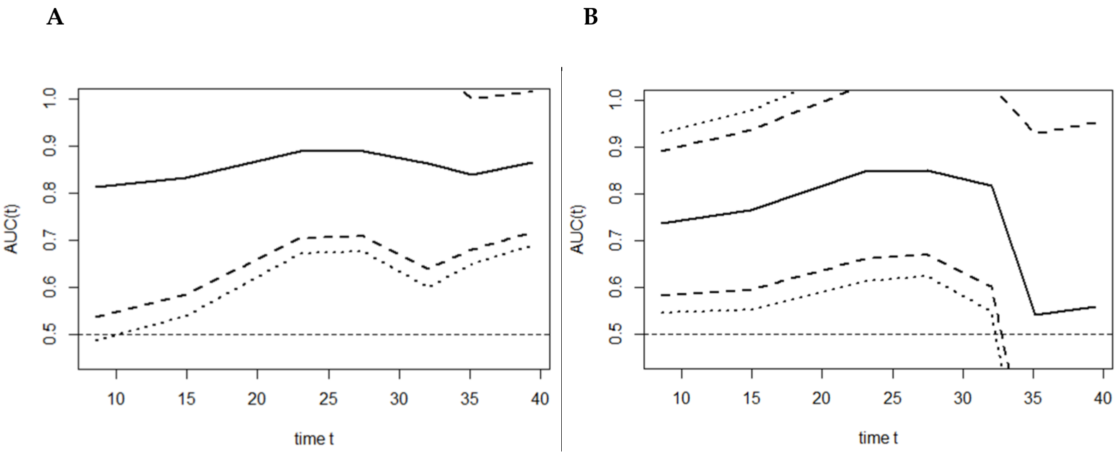

Supplementary Figure 4. The curve of time-dependent area under the curve (AUC) for predicting the overall survival of the H6C score (A) and the Model for End-Stage Liver Disease (MELD) (B) in the validation cohort. The AUCs of the H6C score were consistently higher than those of the MELD over time.

Supplementary Table 1. Baseline characteristics of the study population according to the H6C score.

	**H6C < 45 (*n* =138)**	**45 ≤ H6C<55 (*n* =147)**	**55 ≤ H6C < 64 (*n* =142)**	**64 ≤ H6C (*n* =139)**	***p* value**
Age (year)	51.61±9.81	52.15±9.71	53.94±9.80	52.45±9.45	0.213
Male, no. (%)	107 (77.5)	111 (75.5)	113 (79.6)	108 (74.5)	0.778
Platelet count	132.22±76.06	113.72±74.50	116.54±68.49	106.93±69.67	0.029
Albumin (g/dL)	3.88±0.33	3.47±0.50	3.19±0.47	2.77±0.40	<0.001
Total bilirubin (mg/dL)	0.89±0.46	1.19±0.74	1.68±1.08	2.68±1.20	<0.001
AST (U/L)	69.19±109.43	63.81±43.62	68.50±50.61	71.52±85.27	0.856
ALT (U/L)	62.98±144.46	45.85±77.31	35.73±40.77	32.12±31.11	0.013
PT (INR)	1.08±0.13	1.13±0.14	1.21±0.17	1.44±0.28	<0.001
Creatinine (mg/dL)	0.78±0.20	0.74±0.18	0.77±0.20	0.76±0.24	0.403
Ascites, no. (%)					<0.001
None	130 (94.2)	102 (69.4)	57 (40.1)	11 (7.9)	
Small	8 (5.8)	44 (29.9)	71 (50.0)	77 (55.4)	
Moderate	0 (0)	1 (0.7)	14 (9.9)	51 (36.7)	
Hepatic encephalopathy, no. (%)					<0.001
None	138 (100)	146 (99.3)	130 (91.5)	114 (82.0)	
Grade 1-2	0 (0)	1 (0.7)	9 (6.3)	19 (13.7)	
Grade 3-4	0 (0)	0 (0)	3 (2.1)	6 (4.3)	
Child-Pugh score	5.20±0.40	6.10±0.71	7.20±0.72	9.22±1.26	<0.001
MELD	7.90±1.51	8.76±2.01	10.27±2.53	13.91±2.97	<0.001
HVPG	9.52±2.63	13.17±3.64	15.59±3.66	19.39±4.36	<0.001
H6C score	40.70±2.74	49.74±2.95	58.82±2.54	74.68±8.12	<0.001

Data were presented as mean±SD.

AST, aspartate aminotransferase; ALT, alanine aminotransferase; MELD, model for end stage liver disease; HVPG, hepatic venous pressure gradient, SD, standard deviation.

Supplementary Table 2. Baseline characteristics of the derivation cohort according to the etiologies of chronic liver disease.

	Total (*n* =566)	Viral etiology (*n* =192)	Non-viral etiology (*n* =374)	*p* value
Age (year)	52.55±9.68	51.99±9.60	52.82±9.76	0.334
Male, no. (%)	435 (76.9)	124 (64.6)	311 (83.2)	<0.001*
Platelet count	116.77±72.44	97.71±61.56	127.31±75.87	<0.001*
Albumin (g/dL)	3.32±0.59	3.38±0.67	3.30±0.54	0.146
Total bilirubin (mg/dL)	1.63±1.15	1.45±0.98	1.69±1.20	0.013*
AST (U/L)	67.98±75.94	70.63±96.72	66.94±63.39	0.586
ALT (U/L)	43.92±85.59	63.14±133.79	34.35±41.92	0.004*
Prothrombin time (INR)	1.22±0.26	1.23±0.24	1.20±0.23	0.142
Creatinine (mg/dL)	0.76±0.21	0.76±0.19	0.76±0.21	0.862
Ascites, no. (%)				0.172
None	300 (53.0)	110 (57.3)	190 (50.8)	
Small	200 (35.3)	62 (32.3)	138 (36.9)	
Moderate	66 (11.7)	20 (10.4)	46 (12.3)	
Hepatic encephalopathy, no. (%)				0.079
None	528 (93.3)	184 (95.8)	344 (92.0)	
Grade 1-2	29 (5.1)	6 (3.1)	23 (6.1)	
Grade 3-4	9 (1.6)	2 (1.1)	7 (1.9)	
Child-Pugh score	6.97±1.76	6.71±1.80	7.03±1.65	0.038*
MELD	10.31±3.42	10.09±3.23	10.25±3.26	0.595

Data were presented as mean±SD.

AST, aspartate aminotransferase; ALT, alanine aminotransferase; MELD, model for end stage liver disease; SD, standard deviation.

Supplementary Table 3. Estimated time-dependent AUC of the H6C score and the MELD among the validation cohort.

Time (months)	H6C		MELD	
	AUC	95% CI	AUC	95% CI
8.6	0.814	0.538–1.090	0.739	0.585–0.893
14.9	0.834	0.586–1.081	0.766	0.596–0.937
27.5	0.890	0.710–1.070	0.850	0.670–1.030
35.1	0.841	0.681–1.001	0.542	0.155–0.930
39.4	0.866	0.717–1.015	0.559	0.166–0.952

AUC, area under the receiver operating characteristic curve; MELD, model for end stage liver disease, CI, confidence interval.
